# Man and His Enemies

**Published:** 1889-03-23

**Authors:** 


### The Doctor's Pulpit.

### III.—MAN AND HIS ENEMIES.

### Birth and Babyhood.

In the case of all animals nearly allied to man, that is of all mammals, entrance into the world is accompanied by danger to the offspring as well as to the mother. Structural and constitutional peculiarities in the parent, and aberrations and malformations in the progeny, result in a certain definite proportion of injuries and deaths. After birth the infant enters upon an environment peculiar to itself. The newly-born baby of civilisation is probably at some disadvantage as compared with the offspring of savages and many of the lower animals. It is perhaps undoubted that during the period of motherhood the physical vigour of outdoor labourers, savages, and wild animals is much greater than that of highly civilised women, and that their power of resistance and survival are correspondingly increased. Civilisation, therefore, whilst surrounding infancy with various kinds of protection, has decidedly diminished the natural viability of the human species.

It is passing strange that an apology should seem to be necessary for considering and publicly discussing the breeding and rearing of human beings on exactly the same lines as the breeding and rearing of domestic animals. Yet what is man but an animal, and especially in the earliest stages of his existence? And is he not, at least, to his own race, the most important of all animals? The light of science is shut out from one of the highest regions, and some of its chief labours are wasted, if man alone in his infancy is not to be regarded from the scientific standpoint. Who blames the farmer when he studies carefully every condition, advantageous or disadvantageous, to the production and rearing of valuable horses, cattle, sheep, or even pigs? Is he not blamed for want of study rather, and does not the lack of such study often land him in bankruptcy and poverty? If the baby in arms could for a brief period be placed in the position of a man of forty, it is certain that he would at once pronounce judgment in favour of every consideration and measure in his rearing which would tend to give him the maximum of vigour both of body and mind.

It may be taken for granted that the average infant of civilisation begins life with feebler constitutional resources than those inherited by his savage competitor. That ought not to be so. The civilised man ought to be able to devise methods of living which would give him an advantage over the savage even in inherited physical vigour. To the extent in which he fails there, to that extent he must be held wanting in mental capacity, in real brain power; and to that extent civilisation must be regarded as a failure. Farmers speak of good and bad "strains" in animals. Such an animal is of a "good strain;" such another is of a "bad strain." No amount of skill in nursing and rearing will ever make the animal of the bad strain equal to that of the good strain under like conditions. A good strain gives a creature such a start in the race that, other things being equal, it is sure to come in first at the winning-post. A human child born of a "bad strain," has an enemy within himself more dangerous and injurious than all the ordinary forces of injury which are likely to oppose him in the external world.

"Savage" babies, and babies of out-door workers have generally a decided advantage during the first few months of life, as well as at birth. They are almost invariably suckled and nursed by their own mothers. They are thus fed on a pabulum perfectly suited to their constitution and condition, and that in its most perfect form. It may be argued that the poor feed more coarsely and poorly than the rich, and that therefore their blood and the secretions from it are not so wholesome and nutritious. But it must be remembered that the human mother is an omnivorous feeder, and that however poor her food may be, if she gets enough of it, and lives under the healthy conditions of outdoor life, she is able to convert the coarsest dietary into pure blood and nourishing secretions. One of the most formidable enemies the infant human being has to encounter is, undoubtedly, artificial feeding. Almost as formidable, and in some cases even more so, is the nursing by a weakly or diseased mother. "The blood is the life" of the organism, and food is the life of the blood. But what does the food of the blood consist of? Is digested matter from the stomach its sole source of supply? By no means. One of the most important and essential factors in the food of the blood is pure air. Without this it can never be healthy. With this in abundance, the coarsest food eaten by the mother may be turned into infant nutriment of the most perfect kind.

Artificial feeding, in spite of the risks it involves, is sometimes the only really good thing which can be done for an infant. When such feeding is required the mother herself or other intelligent person should be held responsible for the selection of a proper food. Such food should fulfil the following indispensable conditions: It must be decidedly palatable to the baby. If he makes a wry face or spits it out, it ought to be at once discarded. It should be digestible. If it cause flatulent distension, pain, vomiting, or diarrhœa, it is injurious, and must be changed. It should be nutritious. If the child does not thrive and grow, but pines and looks miserable, the food is poison to him, and must on no account be persevered with. Every infant food is to be regarded as a possible enemy, and should only be continued in use after the most convincing proofs of its agreeableness and value as a nutriment.

A "stuffy" nursery is a pest-house for babies. So far as we can judge nature intended human beings to live constantly in the open air. Our lungs and skin, and other organs are designed for open-air life. It ought to be one of the principal aims of a mother or nurse to keep the air of the nursery as pure as the outside atmosphere. Some inlet for fresh air, some outlet for used-up air should be kept open day and night. When the children go out for their walk the windows and doors are to be thrown wide open. The want of pure air not only pales the infant's cheek but poisons his blood. It helps to make his digestion bad, it prevents him getting sound and refreshing sleep, it modifies and enfeebles the normal actions of the liver and other secreting organs. It, in fact, puts the child out of sorts altogether, and makes him ten times more liable than he should be to catch all kinds of diseases that may be in his vicinity. If the boy were old enough he could seek fresh air for himself; but in the meantime he is perfectly helpless, and at the mercy of every poisonous breeze that blows, and of every tainted atmosphere in which an ignorant mother or a careless nurse may place him.

Changes of temperature within doors and outside are constant sources of danger to the newly-born. Even to grown-up people a fall from 65 deg. to 45 deg. may lead to prompt mischief. But if that be so with adults, what must it be to the sensitive and fragile creatures whom a tumble on the floor might kill in a single moment. The natural heat, vigour, and resisting power of infants is of necessity exceedingly small; is, indeed, strictly comparable to the strength of their limbs, which is almost nil. Yet how often it is considered that an infant may be allowed to run any risk, however great, from change of temperature, so long as it makes no crying complaint! The temperature of the night nursery must be a few degrees lower than that of the day nursery or play-room, though the difference is not to be too great. It is very desirable, particularly during the day, to keep the temperature at the same level. To sometimes have the fire nearly out, and at other times to have it large enough to roast half a bullock, is the very essence of stupidity and wicked carelessness. If the present writer could be a baby again he would like nothing better than to put a charge of gunpowder under a lazy or novel-reading nurse's chair, and to blow her up to the ceiling at the very moment when she is in the midst of her most interesting chapter.

The most dangerous of all enemies to infants are unthinking mothers and uncaring nurses. The appeal of a helpless baby to a woman's heart is, one would think, irresistible. But what will make a thoughtless woman thoughtful, or a heartless nurse kind and gentle? Most of the stomach disorders, the diarrhœas, the pleurisies, the bronchitis, the pneumonia, and other like troubles which children suffer and die from, are the result of carelessness and incapacity on the part of parent or nurse. If all the nursemaids were to be hanged whose recklessness is the cause of infant deaths, Mr. Berry would never need occupation. Even mothers cannot hold themselves guiltless. They often leave to nurses what they should do themselves, and not seldom are the indirect cause of children's deaths. Poor babies! Providence sends them into the world in the full confidence that their touching and pathetic helplessness will win all hearts, and secure for them that daily care and protection which are indispensable to health and life. How worthless and inhuman must be those hearts which fail to respond to an appeal so irresistible in its utter powerlessness!

*(To be continued.)*

